# Education and Educational Institutions Considered, with Reference to the Industrial Professions and the Present Aspect of Society

**Published:** 1847-07

**Authors:** 


ART. VI.—*Education and Educational Institutions considered, with reference to the Industrial Professions and the present aspect of Society.* By the Rev. J. BOOTH, LL.D., F.R.S., M.R.I.A., &c., formerly Principal of Bristol College, and Vice-Principal of the Liverpool Collegiate Institution.—*London,* 1846. 8vo, pp. 108.

WE commend this essay to the special consideration of all who are interested in the subject of which it treats,—and who among our readers is not?—the education of the middle classes. It might be almost regarded as an expansion of our own article on the Preliminary Education of the Medical Student (Vol. IX); and did we not happen to know that Dr. Booth was unacquainted with that article, until after the publication of his treatise, we might have got up a very plausible charge of plagiarism against him. We need scarcely add, therefore, that Dr. Booth's views, expressed in a clear logical style, with much felicity of illustration, have our hearty concurrence upon almost every point; and they are, of course, the more valuable, as coming from a gentleman of greater practical acquaintance with educational matters than falls to the lot of most men. Did our space permit, we should have been glad to present our readers with a few extracts, which point out, in a very striking manner, the insufficiency of the system of education generally pursued in our public schools to meet the wants of the present age, and the importance of substituting the study of the exact sciences for a portion of the very imperfect though prolonged course of instruction now given in classics. Upon these points, however, we cannot now dwell; nor shall we grapple with the question of the degree in which the Government of this country ought to support and regulate such institutions as those with which Dr. Booth has been connected. We must remark, however, that we cannot but regret to find Dr. Booth still so far trammelled by class prejudices, as to give what we must regard an unfair representation of the leading doctrine of the 'Vestiges of Creation' (p. 69, *note*). His acquaintance with philosophical history, however, has enabled him to prove the curious fact—that the general idea was very clearly stated by Seneca; an "anticipation" which was not a little remarkable in the then state of science.

